# A comparison of muscle strength and endurance, exercise capacity, fatigue perception and quality of life in patients with chronic obstructive pulmonary disease and healthy subjects: a cross-sectional study

**DOI:** 10.1186/1471-2466-14-6

**Published:** 2014-01-27

**Authors:** Ebru Calik-Kutukcu, Sema Savci, Melda Saglam, Naciye Vardar-Yagli, Deniz Inal-Ince, Hulya Arikan, Zeynep Aribas, Ozge Ozer, Meral Bosnak-Guclu, Lutfi Coplu

**Affiliations:** 1 Department of Physiotherapy and Rehabilitation, Faculty of Health Sciences, Hacettepe University, Samanpazari, Ankara 06100, Turkey; 2 School of Physiotherapy and Rehabilitation, Dokuz Eylul University, Izmir, Turkey; 3 Department of Physiotherapy and Rehabilitation, Faculty of Health Sciences, Gazi University, Ankara, Turkey; 4 Department of Chest Medicine, Faculty of Medicine, Hacettepe University, Ankara, Turkey

**Keywords:** COPD, Exercise tests, Life quality, Muscle, Strength, Muscular fatigue, Coughs

## Abstract

**Background:**

Chronic obstructive pulmonary disease (COPD) has significant systemic effects that substantially impact quality of life and survival. The purpose of this study was to assess and compare peripheral muscle strength and endurance, exercise capacity, fatigue perception and quality of life between patients with COPD and healthy subjects.

**Methods:**

Twenty COPD patients (mean FEV_1_ 49.3 ± 19.2%) and 20 healthy subjects were included in the study. Pulmonary function testing and six-minute walk test (6MWT) were performed. Peripheral muscle strength was measured with a hand-held dynamometer, peripheral muscle endurance was evaluated with sit-ups, squats and modified push-ups tests. Fatigue perception was assessed using the Fatigue Impact Scale (FIS) and Fatigue Severity Scale (FSS). General quality of life was determined with the Nottingham Health Profile (NHP), and cough-specific quality of life was evaluated with the Leicester Cough Questionnaire (LCQ).

**Results:**

Pulmonary functions, strength of shoulder abductor and flexor muscles, numbers of sit-ups and squats, 6MWT distance and 6MWT% were significantly lower in COPD patients than in healthy subjects (p < 0.05). FIS psychosocial sub-dimension and total scores, NHP scores for all sub-dimensions except pain sub-dimension of the COPD group were significantly higher than those of healthy subjects (p < 0.05). The LCQ physical, psychological and social sub-dimensions and total scores were significantly lower in COPD patients than in healthy subjects (p < 0.05).

**Conclusions:**

Pulmonary functions, peripheral muscle strength and endurance, exercise capacity and quality of life were adversely affected in patients with COPD. There are greater effect of fatigue on psychosocial functioning and general daily life activities and effect of cough on the quality of life in patients with COPD. This study supports the idea that COPD patients must be evaluated in a comprehensive manner for planning pulmonary rehabilitation programs.

## Background

Chronic obstructive pulmonary disease (COPD) is one of the main causes of morbidity and mortality worldwide. COPD is expected to be fourth in the causes of death and seventh in the causes of loss of Disability-Adjusted Life Years (DALY) worldwide by 2030 [1]. The disease is often accompanied by concomitant systemic manifestations leading to a deterioration in functional capacity, worsening dyspnea and decreased health-related quality of life, and increased mortality [2].

Skeletal muscle weakness is one of the major systemic effects of COPD and associated with the loss of lean body mass [2,3]. When comparing isometric quadriceps strength between patients with COPD and age-and-sex-matched healthy controls, a 20-30% loss in muscle strength and a tendency to fatigue in the muscle were found [4]. Seven controlled studies in COPD reported a loss of muscular endurance greater than that of skeletal muscle strength [2]. The limitations to exercise in COPD reflect the abnormalities of the respiratory, cardiovascular, neuromuscular and neurosensory systems in highly variable combinations with multifactorial interactions [5]. Exercise tolerance as assessed by both a maximal exercise test and a 6-minute walk test (6MWT) was shown to be lower than in people with COPD than in sedentary healthy subjects [6].

Fatigue is one of the most distressing symptoms of this group of diseases; it significantly impacts both functional performance and quality of life [7-9]. Studies have shown that skeletal muscle fatigue limits exercise tolerance in 50% of patients; fatigue was found to be increased in patients when compared to healthy controls [2,10-12]. The physical and psychosocial effects of cough, a characteristic symptom of COPD, may lead to a worsening of the quality of life [1,13-15].

Even though it has been reported that the systemic effects of COPD impair peripheral muscle strength and endurance, exercise tolerance and quality of life, studies about practical evaluation methods for the peripheral muscle endurance and perception of fatigue in pulmonary rehabilitation programs of patients with COPD are limited. There is no study that show the impairments of COPD than healthy people with practical assessment methods in pulmonary rehabilitation. While published studies exist comparing COPD with other similar disease groups for the effect of cough on quality of life, there were not found any study comparing it with healthy subjects. Therefore, this study aimed to evaluate peripheral muscle strength and endurance, exercise capacity, fatigue perception and both general and cough-specific quality of life and to compare these results with those of a group of healthy subjects. The primary objective of this study was to show how much the systemic effects influence patients with COPD than healthy subjects. The secondary objectives of the study were to show discriminative properties of practical evaluation methods of systemic effects of COPD than healthy controls and to investigate relationships between dyspnea and fatigue perception, peripheral muscle strength, general and cough-specific quality of life in patients with COPD.

## Methods

Twenty patients with COPD between the ages of 40–80 who were not taking antibiotics and had not made any changes in their medications for at least three weeks (mean FEV_1_ 49.3 ± 19.2%, 5 female and 15 male) and a group of age-and-sex-matched healthy subjects (4 female and 16 male) were included in this cross-sectional study. The patients who were redirected from the Hacettepe University Hospitals, Department of Chest Diseases, Ankara, Turkey for pulmonary rehabilitation and willing to participate in the study were recruited. The healthy subjects were volunteers without any lung, heart or systemic disease or handicap in physical effort. Patients who have physical disability to perform the tests and could not cooperate for the tests were not included in the study. The assessments were applied in the Hacettepe University, Faculty of Health Sciences, Department of Physiotherapy and Rehabilitation, Cardiopulmonary Rehabilitation Unit, Ankara Turkey. The study was approved by the Ethical Committee of Hacettepe University, patients and healthy subjects who decided to participate in the study signed an informed consent form.

The physical and sociodemographic characteristics of all subjects were recorded. Dyspnea levels of subjects were evaluated using the Modified Medical Research Council (MMRC) Dyspnea Scale. This is a categorical scale ranging from 0 to 4, where patients choose the statement that best describes their dyspnea level from five choices [16].

The pulmonary function test according to American Thoracic Society/European Respiratory Society (ATS/ETS) criteria was performed with a Spirolab III spirometer (Spirolab, Medical International Research, Rome, Italy) in the sitting position. The pulmonary function test results were expressed as percentages of the expected values adjusted for age, height, body weight and sex [17].

Peripheral muscle strength (knee extensor, shoulder abductor and flexor, and hand grip strength) was measured using a digital hand-held dynamometer (JTECH, Medical Commander Powertrack II, USA). Peripheral muscle strength testing was repeated three times for each muscle and the mean value in Newtons (N) was recorded. The average values of the left and right sides was then taken for statistical analysis. Peripheral muscle endurance was assessed with a sit-ups test, a modified push-ups test and a squat test. In the sit-ups test, patients were asked to lift up the trunk from the supine position until the lower the angle of the scapula with the arms stretched forward consecutively as quickly as possible. For the modified push-ups test, patients were requested to only lift their trunk performing consecutive elbow flexions and extensions in the push-up position as quickly as possible. The squat test required the patient to move as quickly as possible from a standing position to a squatting position. Each tests were performed for 30 seconds and the numbers of sit-ups, push-ups and squats were recorded for analysis [18].

For the 6MWT, the patients were requested to walk along a flat corridor as fast as they could in their walking speed for six minutes. The test was administered twice in the same day with a half-hour interval. Pre- and post-test heart rate (HR) and oxygen saturation (SpO_2_) with a pulse oxymeter (KPTS, Seoul, Korea) were recorded [19]. General and quadriceps fatigue and dyspnea perception were assessed with Modified Borg Scale and pre- and post-test scores were recorded [20]. For each patient, result of the tests with the longest distance was used in the statistical analysis [19]. The 6MWT distance was expressed as percentages of the expected values from age and sex (6MWT% of distance) [21].

After physical assessments, the patients and healthy subjects answered questionnaires about fatigue and quality of life by themselves. The subjects answered questionnaires completed by selecting the most suitable option for them. The fatigue perception of the subjects were evaluated by the Fatigue Impact Scale (FIS) and the Fatigue Severity Scale (FSS). FIS is a multidimensional scale consisting of 40 questions to evaluate the patient’s perception of the limitations caused by fatigue during the last month in the physical (10 items), cognitive (10 items) and psychosocial (20 items) functions. Each question is answered by a choice ranging from a score of 0 (no problem) to 4 (extreme problems). Total score ranges between 0 and 160. High scores indicate a higher effect of fatigue [22]. FSS is one of the most frequently used 9-item one-dimensional scale developed for evaluating fatigue. Patients are asked to provide a score for each item on a range from 1 (strongly disagreement) to 7 (strongly agreement), a score of ≥4 indicates severe fatigue. FSS is reliable and valid assesment tool for the Turkish population [23].

The Nottingham Health Profile (NHP) is a reliable general quality of life questionnaire developed to provide a summary index on the patient’s perception of emotional, social and physical health problems and adaptation of the NHP was made for the Turkish people (24). In this study, only the first part of this questionnaire was used to evaluate the subjects’ general quality of life. This first part contains 38 items evaluating the perception of limitation or discomfort, divided over six sub-dimensions: energy level (3 items), pain (8 items), emotional reaction (9 items), sleep (5 items), social isolation (5 items), and physical abilities (8 items). The total score for each section is 0–100. High scores indicate worse quality of life [24].

The Leicester Cough Questionnaire (LCQ) is a valid and reliable health status measure for adults with chronic cough and patients with COPD that indicates the effect of cough on quality of life and the efficacy of cough-modifying agents during the last two weeks. Adaptation of the LCQ was made for the Turkish people (25). It contains a total of 19 items that are divided into the physical (8 items), psychosocial (7 items) and social (4 items) sub-dimensions. The answer to each items is scored 1–7. Total score varies from 3 to 21. Low scores on the LCQ indicate a higher effect of coughing on the subject [25,26].

The statistical evaluation was performed using the SPSS 15.0 statistical packet software for Windows [27]. Variables were descriptively expressed as mean ± standard deviation, frequency and percentage. Normal distribution was evaluated by the Shapiro-Wilk test and histograms and the assumptions of the parametric tests were met by the data. The continuous variables were compared using the two-tailed Student’s *t*-test and variables determined by counting were compared with the two-tailed Chi-squared test. Correlations between dyspnea and fatigue perception, peripheral muscle strength, general and cough-specific quality of life in patients with COPD were evaluated using the two-tailed Spearman’s correlation analysis as the conditions required for parametric tests were not fulfilled [28]. Correlations were characterized as "high" (r > 0.70), "moderate" (r = 0.50–0.69), "low" (r = 0.26–0.49) and "little or no correlation" (r = 0.00–0.25) [29]. Significance was set at α < 0.05.

## Results

### Pulmonary function tests and dyspnea levels

The subjects’ characteristics are shown in Table 1. Physical and demographic characteristics of patients with COPD and healthy subjects were similar. FVC, FEV_1_, FEV_1_/FVC, FEF_25-75%_ and PEF values of patients were significantly lower than those of healthy subjects (p = 0.001, Table 1). According to the Global Initiative for Chronic Obstructive Lung Disease (GOLD) criteria, 5% of patients had mild, 45% moderate, 30% severe and 20% very severe COPD [1]. The cigarette consumption (p = 0.024) and dyspnea levels (p = 0.001) were significantly higher in COPD patients than in the healthy subjects (Table 1).

**Table 1:** Characteristics of COPD and healthy subjects

**Characteristics**	**COPD (n = 20)**	**Healthy (n = 20)**	**p**
Age (years)	53 ± 5 [%95 Cl 52–56]	51 ± 6 [%95 Cl 47–54]	0.125
Sex (male/female)	15/5	16/4	1.0
Height (cm)	167±9 [%95 Cl 163–172]	166 ± 7 [%95 Cl 163–169]	0.496
Body weight (kg)	79 ± 18 [%95 Cl 69–86]	80 ± 10 [%95 Cl 72–82]	0.77
Body mass index (kg/m^2^)	28 ± 5 [%95 Cl 25–30]	27 ± 4 [%95 Cl 26–30]	0.389
FVC (%)	66 ± 19 [%95 Cl 56–75]	99 ± 13 [%95 Cl 91–105]	0.001*
FEV_1_ (%)	49 ± 19 [%95 Cl 39–58]	98 ± 12 [%95 Cl 90–103]	0.001*
FEV_1_/FVC (%)	63 ± 13 [%95 Cl 56–68]	82 ± 5 [%95 Cl 79–85]	0.001*
FEF_25-75%_ (%)	28 ± 17 [%95 Cl 19–33]	89 ± 22 [%95 Cl 75–100]	0.001*
PEF (%)	57 ± 22 [%95 Cl 45–65]	105 ± 21 [%95 Cl 95–118]	0.001*
Smoking history (pack-years)	30 ± 24 [%95 Cl 19–42]	13 ± 15 [%95 Cl 5–22]	0.024*
MMRC (0–4)	1.4 ± 1.0 [%95 Cl 1–2]	0.3 ± 0.6 [%95 Cl -0.1-56]	0.001*

*Student’s *t*-test, p < 0.05.

*Abbreviations*: FVC forced vital capacity, FEV_1_ forced expiratory volume in one second, FEF_25-75%_ forced expiratory flow 25–75%, PEF peak expiratory flow, MMRC Modified Medical Research Council dyspnea scale.

### Peripheral muscle strength and endurance

Shoulder abductor (p = 0.012) and flexor muscle strengths (p = 0.027) were also significantly lower for patients than for healthy subjects (Table 2). There was no statistically significant difference for knee extensor (p = 0.624) and hand grip strengths (p = 0.523) between two groups (Table 2). The numbers of sit-ups (p = 0.019) and squats (p = 0.009) were significantly lower in the COPD patients than in the healthy subjects but no significant difference was found between two groups regarding the numbers of modified push-ups (p = 0.065, Table 2).

**Table 2:** Peripheral muscle strength and endurance, exercise capacity in patients with COPD and healthy subjects

	**COPD (n = 20)**	**Healthy (n = 20)**	**p**
Shoulder abductors (N)	147 ± 38 [%95 Cl 135–171]	183 ± 48 [%95 Cl 161–206]	0.012*
Shoulder flexors (N)	168 ± 53 [%95 Cl 147–199]	204 ± 45 [%95 Cl 183–225]	0.027*
Knee extensors (N)	312 ± 54 [%95 Cl 277–336]	320 ± 44 [%95 Cl 300–341]	0.624
Hand grip strength (N)	185 ± 34 [%95 Cl 158–195]	195 ± 56 [%95 Cl 168–221]	0.523
Sit-ups (n)	16 ± 5 [%95 Cl 13–18]	19 ± 4 [%95 Cl 17–21]	0.019*
Squats (n)	17 ± 3 [%95 Cl 15–18]	20 ± 4 [%95 Cl 18–22]	0.009*
Modified push-ups (n)	17 ± 5 [%95 Cl 14–20]	19 ± 4 [%95 Cl 18–21]	0.065
6MWT distance (m)	549 ± 92 [%95 Cl 526–611]	620 ± 60 [%95 Cl 592–648]	0.006*
6MWT%	98 ± 16 [%95 Cl 95–109]	111 ± 9 [%95 Cl 107–115]	0.002*
HR max. %	71 ± 13 [%95 Cl 63–79]	70 ± 8 [%95 Cl 67–74]	0.708
ΔSpO_2_ (%)	-3.4 ± 6.0 [%95 Cl -5-0.3]	0.2 ± 1.4 [%95 Cl -0.5-0.8]	0.016*
Modified Borg-dyspnea (0–10)	2.7 ± 2.8 [%95 Cl 1.1-3.9]	0.1 ± 0.3 [%95 Cl -0.0-0.2]	0.001*
Modified Borg-fatigue (0–10)	1.0 ± 2.4 [%95 Cl -1.1-2.8]	0.4 ± 0.6 [%95 Cl 0.1-0.6]	0.245
Modified Borg-quadriceps fatigue (0–10)	2.6 ± 2.1 [%95 Cl 1.6-3.9]	0.3 ± 0.8 [%95 Cl -0.1-0.7]	0.001*

*Student’s *t*-test, p < 0.05.

*Abbreviations:* 6MWT six minute walk test, HR heart rate, SpO_2_ oxygen saturation.

### Exercise capacity

The 6MWT distance (p = 0.006) and 6MWT% distance (p = 0.006) were significantly lower in patients with COPD than in the healthy subjects (Table 2). The changes in SpO_2_ (p = 0.016), dyspnea (p = 0.001) and quadriceps fatigue (p = 0.001) during 6MWT were significantly higher in patients than healthy subjects (Table 2). No statistically significant difference was noted between groups for percentage of maximal HR (p = 0.708) or general fatigue perception (p = 0.245) recorded during the test (Table 2).

### Fatigue perception

Eleven patients (55%) with COPD reported having severe fatigue. The FIS total (p = 0.048) and psychosocial sub-dimension (p = 0.046) scores of patients with COPD were higher in than those of healthy subjects, while there was no statistically significant difference for the FIS physical (p = 0.058) and cognitive sub-dimensions (p = 0.064) and FSS scores (p = 0.503, Table 3).

**Table 3:** Fatigue perception, general and cough-specific quality of life in patients with COPD and healthy people

	**COPD (n = 20)**	**Healthy (n = 20)**	**p**
**FIS**			
Physical	12 ± 11 [%95 Cl 7–17]	6 ± 8 [%95 Cl 2–10]	0.058
Cognitive	9 ± 8 [%95 Cl 5–13]	5 ± 7 [%95 Cl 1–8]	0.064
Psychosocial	20 ± 17 [%95 Cl 12–27]	97 ± 15 [%95 Cl 2–16]	0.046*
Total	41 ± 35 [%95 Cl 24–57]	20 ± 29 [%95 Cl 6–33]	0.048*
**FSS**	4 ± 1 [%95 Cl 3–5]	3 ± 2 [%95 Cl 2–4]	0.503
**NHP**			
Energy level	43 ± 43 [%95 Cl 23–63]	8 ± 21 [%95 Cl -1-18]	0.03*
Pain	23 ± 28 [%95 Cl 10–35]	9 ± 14 [%95 Cl 2–15]	0.056
Emotional reaction	33 ± 27 [%95 Cl 20–46]	12 ± 19 [%95 Cl 3–22]	0.009*
Sleep	31 ± 29 [%95 Cl 17–44]	11 ± 19 [%95 Cl 2–19]	0.015*
Physical abilities	24 ± 21 [%95 Cl 14–34]	9 ± 12 [%95 Cl 3–15]	0.011*
Social isolation	27 ± 35 [%95 Cl 11–43]	5 ± 18 [%95 Cl -4-13]	0.018*
Total	180 ± 144 [%95 Cl 113–248]	51 ± 62 [%95 Cl 22–80]	0.001*
**LCQ**			
Physical	5.3 ± 1.3 [%95 Cl 5–6]	6.7 ± 0.7 [%95 Cl 6–7]	0.001*
Psychological	5.2 ± 1.2 [%95 Cl 5–6]	6.1 ± 0.4 [%95 Cl 5.9-6.2]	0.006*
Social	5.7 ± 1.5 [%95 Cl 5–6]	6.9 ± 0.3 [%95 Cl 6.7-7]	0.004*
Total	16.3 ± 3.8 [%95 Cl 12–17]	19.6 ± 1.3 [%95 Cl 19–20]	0.001*

*Student’s *t*-test, p < 0.05.

*Abbreviations:* FIS Fatigue Impact Scale, FSS Fatigue Severity Scale, NHP Nottingham Health Profile, LCQ Leicester Cough Questionnaire.

### Quality of life

The NHP energy level (p = 0.03), emotional reaction (p = 0.009), physical abilities (p = 0.011) and social isolation (p = 0.018) sub-dimensions and the total scores (p = 0.001) were significantly higher in COPD patients than in healthy subjects (Table 3). The LCQ physical (p = 0.001), psychological (p = 0.006) and social (p = 0.004) sub-dimensions and total scores (p = 0.001) of patients were significantly lower than those of healthy subjects (Table 3).

### Correlation analysis

**Figure 1:**
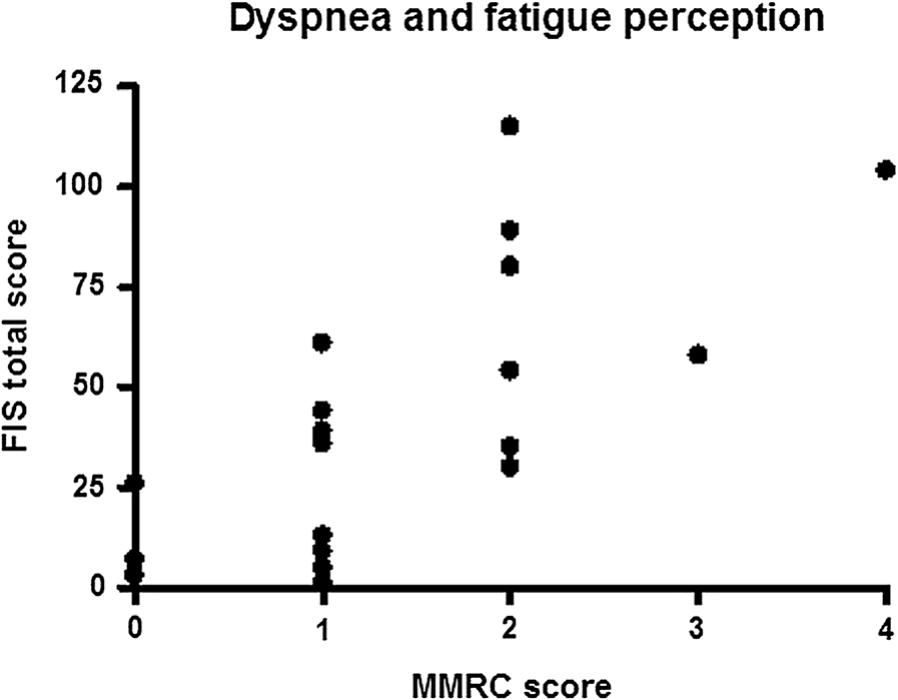
The relationship between the MMRC and FIS total scores.

**Figure 2:**
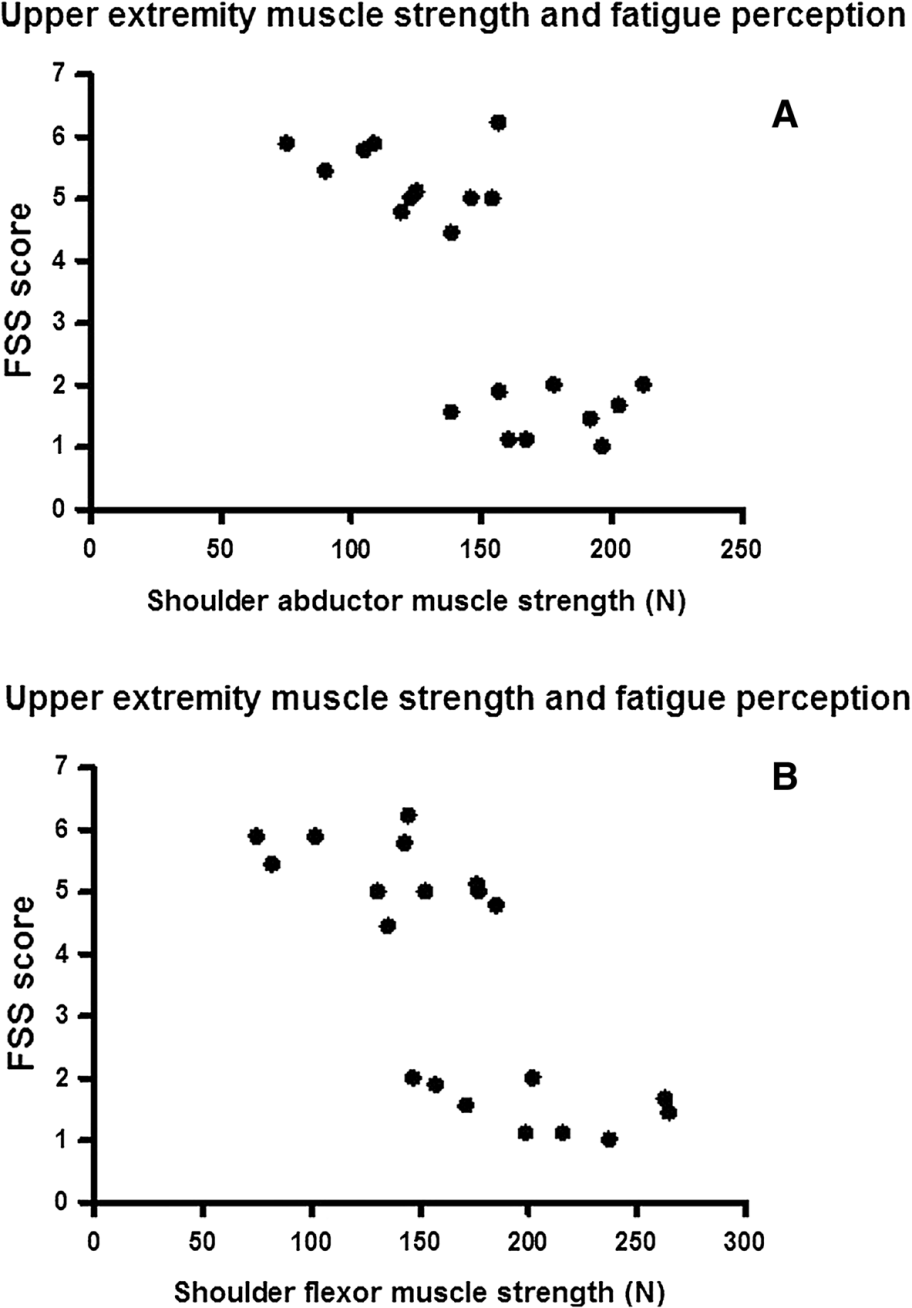
**The relationship between upper extremity muscle strength and fatigue perception. (A)** The shoulder abductors’ strength and FSS score. **(B)** The shoulder flexors’ strength and FSS score.

Correlation analysis showed that the MMRC score was significantly related with scores of the FIS physical (p = 0.001, r = 0.70), cognitive (p = 0.002, r = 0.66), and psychosocial sub-dimensions (p = 0.001, r = 0.72), the FIS total score (p = 0.001, r = 0.69) (Figure 1) and the FSS total score (p = 0.001, r = 0.78). A high degree of correlation was seen between the FSS total score and shoulder abductor strength (p = 0.001, r = -0.72) (Figure 2A), the FSS total score and shoulder flexor strength (p = 0.001, r = -0.78) (Figure 2B).

**Figure 3:**
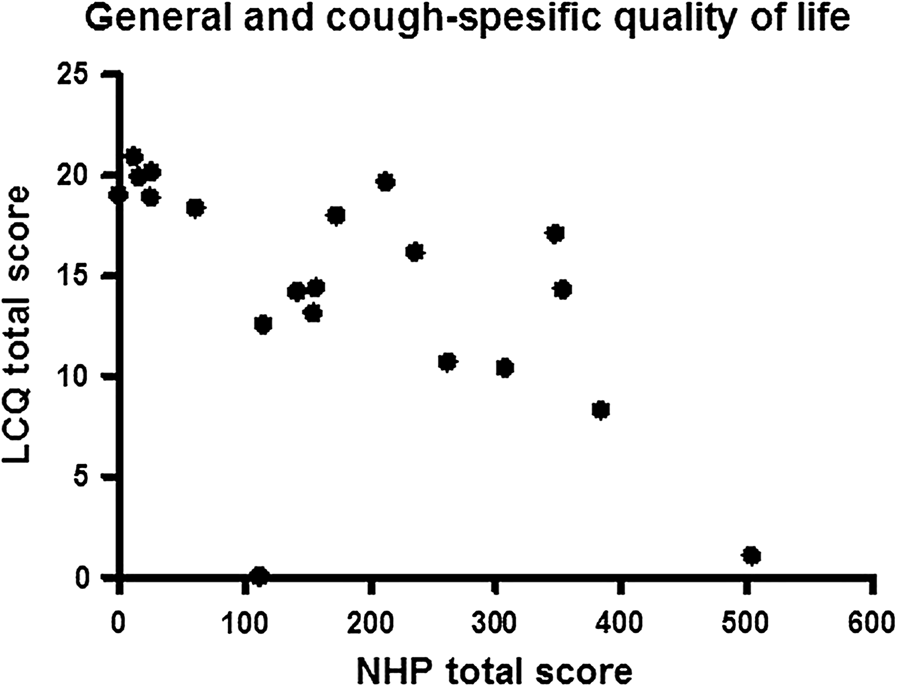
The relationship between the NHP and LCQ total scores.

The NHP sleep sub-dimension score was highly correlated with the LCQ total score (p = 0.001, r = -0.71). The NHP physical abilities sub-dimension score was significantly related with the LCQ physical (p = 0.007, r = -0.58), psychological (p = 0.002, r = -0.65) and social sub-dimensions (p = 0.007, r = -0.59) and total scores (p = 0.001, r = -0.67). The NHP total score was likewise in a statistically significant correlation with the LCQ total score (p = 0.003, r = -0.63) (Figure 3) and scores of its following sub-dimensions: physical (p = 0.013, r = -0.55), psychological (p = 0.008, r = -0.58), and social (p = 0.02, r = -0.52).

## Discussion

This study shows that upper extremity muscle strength, abdominal and quadriceps muscle endurance and exercise capacity were reduced compared to healthy subjects in a group of patients with COPD mostly in GOLD stages II and III. It also indicates that fatigue affects generally the daily life activities and psychosocial life of patients with COPD more negatively than healthy subjects, the general quality of life are deteriorated in the patients. The upper extremity muscle strength and dyspnea perception were found to be associated with fatigue perception in COPD. The importance of this study lies in its being the first to show that cough, a major symptom in COPD patients, have a negative impact on the aspects of physical, psychologic and social life of patients, leads to a worsening of their quality of life as compared to healthy subjects. The loss in cough-specific quality of life was also shown to be related with that in general quality of life.

### Dyspnea perception

Hyperinflation reduces inspiratory capacity and increases functional residual capacity in COPD. This leads to an increase in the dyspnea perception during exercise and a limitation of exercise capacity [1,13]. Higher MMRC and dyspnea scores during 6MWT of our patients with COPD are characteristic of the disease.

### Peripheral muscle strength

Gosselink *et al*. observed a significant decline in peripheral muscle strength when compared 40 patients with moderate to severe COPD (average FEV_1_ 41 ± 19%) with healthy controls. In the COPD group, hand grip and elbow flexor strength were significantly higher than the shoulder abductor strength and knee extensor muscle strength was not different from hand grip and shoulder abductor strength. It has been concluded that muscle weakness does not affect all muscle groups similarly and that peripheral muscle strength is not related with the severity of airway obstruction in patients with stable COPD [30]. It has been reported that unsupported arm exercises result in a greater level of dyspnea than lower extremity exercises even at low workloads, because they interfere with the contribution of accessory inspiratory muscles to the breathing work [31]. It has been thought that the relative weakness of upper extremity muscles in COPD patients compared to healthy controls in this study can be explained with that patients who were mostly at GOLD stages II and III may have limited arms movements in order to prevent dyspnea. The similar hand grip strength in both groups is compatible with the report that distal upper extremity muscles are less affected than proximal muscles in patients with moderate to severe COPD [30]. Even though hand-held dynamometers provide reliable test results, the tester’s strength is particularly important in determining the strength of large muscle groups and has a potential for underestimating maximal muscle strength [32]. Published reports in the literature indicate that quadriceps strength is an important index of exercise capacity in COPD [33]. Differences in the test methods, the absence of similarity in the distribution of muscle weakness in the upper vs. the lower extremity in patients with COPD, the finding that the patients’ exercise capacity had reached up to 98% of the expected values and the similar body compositions in both groups can explain the similarity of quadriceps strength in COPD patients and healthy volunteers in our study.

### Peripheral muscle endurance and exercise capacity

Allaire *et al.* have shown that isometric quadriceps endurance was significantly reduced in 29 COPD patients with severe airway obstruction comparison to its value in healthy subjects [34]. We found that numbers of squats in 30 seconds, which indicate quadriceps endurance, were reduced compared to the healthy subjects in accordance with the published reports [6,34-36]. As a result of motivational factors influencing peripheral muscle endurance measurements and the heterogeneous distribution of musculoskeletal dysfunction in COPD, data on peripheral muscle function reported in the literature is variable. The loss of endurance in the abdominal muscles of COPD patients may be due to deconditioning secondary to inactivity. It has shown that the mechanic efficiency of submaximal arm exercises was substantially unchanged compared to controls and the upper extremity exercise tolerance was clearly higher than that of the lower limbs in patients with stable COPD [37]. In conformity with this finding, our study also showed that the upper extremity muscle endurance, as indicated by the modified push-ups test, was maintained in the patients. Our study showed the feasibility of using these endurance tests to differentiate effects on regional endurance that may be easier to apply in the clinic than other published endurance evaluation methods in COPD. Even though COPD patients underperformed with respect to the controls at the end of 6MWT, they nonetheless remained within normal limits. The finding that exercise capacity remained normal in these cases with preserved quadriceps strength [38] supports the idea that skeletal muscle weakness plays an important role in exercise limitation [33].

### Fatigue perception

Lewko *et al.* have shown that the subjective perception of fatigue in COPD patients, as evaluated by the Multidimensional Fatigue Inventory (MFI-20), was substantially higher than in healthy people [39]. It has been reported in a study of 151 patients with COPD that patients report fatigue as being more frequent, in longer duration and greater severity and that their physical and psychosocial limitations due to fatigue, as assessed by FIS, are relatively greater than people in the general population [8]. Our study indicated that majority of patients with COPD have severe fatigue and fatigue affects the general daily life activities and psychosocial life of patients definitely more than those of healthy subjects. This finding is compatible with the reports in the literature about fatigue in COPD [8,39,40]. Theander *et al*. have found that COPD patients reporting severe fatigue exhibit more pronounced fatigue-related functional limitations than those patients who are reporting moderate fatigue [8]. The finding that there is no significant difference between the groups for the FIS sub-dimensions that assess fatigue-related limitations in physical and cognitive functions is thought to be due to the majority of the patients in GOLD II-III stage and their mild and similar fatigue severity level (mean FSS scores <4) with controls. Mental functions are one of the extremely complex brain functions and their evaluation must be multidimensional. The cognitive sub-dimension of FIS provides a gross assessment of fatigue effect on cognitive function and it may have been insufficient for the evaluation of influence of fatigue on cognitive functions.

In a study in patients with moderate and severe COPD, there was found a high correlation between the levels of fatigue and dyspnea (r = 0.74, p < 0.001) in patients reporting moderate fatigue. Dyspnea, depressive mood and subjective sleep quality were showed to explain 42% of the change in fatigue. The authors explained the relationship between dyspnea and fatigue by the hypothesis that COPD-related fatigue results from an increase in respiratory effort and from physical deconditioning [9]. Even though the mechanisms of the relationship are still unclear, our findings show the presence of a correlation between dyspnea and fatigue in conformity with existing studies in this field [9,41]. Lewko *et al*. find that depression, muscle strength and exercise desaturation explain 62% of the variability in the MFI-20 general fatigue sub-dimension score [39]. The high degree of correlation between peripheral muscle strength and the severity of fatigue in our study supports this finding and pulmonary rehabilitation programs should also consider peripheral muscle weakness beside dyspnea-reduction approaches in the treatment of fatigue. This study guides to literature by showing that the FIS and FFS questionnaires are more practical, easier to use compared to physiological fatigue assessment methods and distinctive for fatigue evaluation.

### Quality of life

The negative effects of COPD on multiple aspects of general quality of life, as evaluated by NHP, supports the published data that indicate a lower quality of life in COPD patients when compared to other chronic disease and healthy subjects [11,42,43]. Studies on chronic respiratory symptoms shows a chronic cough and/or sputum production prevalence in the general adult population varying from 4% in nonsmokers to 50% in patients with COPD [44]. It has been suggested that patients who complain from chronic cough are generally affected by psychosocial complications as well as physical and psychosocial adverse effects [14,15]. Chronic cough and sputum production in COPD patients are related with loss of lung function and increased frequency of exacerbations which more severely worsen quality of life [44]. The cough-specific quality of life of patients with COPD complaining from chronic cough in this study (of whom 10% had non-productive and 50% productive cough) were significantly more severely affected than healthy subjects who reported a history of smoking and complaint from acute productive cough (10% of healthy subjects). This findings supports the published reports which show that cough affects quality of life in COPD patients by leading to physical and psychosocial complications, and the effect on psychosocial aspect of quality of life is more severe in patients with chronic cough than in healthy subjects complaining from acute cough [15,42,45]. Health-related quality of life questionnaires are the best method for expressing cough intensity from the patient’s viewpoint, subjective responses by the patients inform about the frequency and intensity of the cough [14]. The study also shows that the multidimensional LCQ questionnaire which is valid, reliable and responsive instrument [26], may be used as a distinctive questionnaire for evaluating the effect of cough on quality of life.

French *et al*. have demonstrated that the physical and psychosocial adverse effects of chronic, persistent cough have significant psychosocial and physical effects on general quality of life as assessed by the Sickness Impact Profile (SIP), in particular in the categories of ambulation, social interaction, sleep and rest, work, home management, recreation and pastimes. A multiple stepwise regression analysis showed that cough-related tiredness explained approximately 30% of the variability in SIP psychosocial sub-dimension and total scores, and that the need for reassurance that nothing was seriously the matter related to cough, stopped going to the movies and spouse unable to tolerate the cough explained 40% of the variability in the SIP physical sub-dimension score [15]. Another study performed in 54 patients with COPD found significant correlations between the corresponding sub-dimensions of the LCQ and the Saint George’s Respiratory Questionnaire [26]. The findings that moderate and high correlations between the sleep and physical abilities sub-dimensions of the NHP and the corresponding sub-dimensions of the LCQ in this study support the observations showing that cough is related with general quality of life by leading to physical complications and sleep disturbances. While health-related quality of life questionnaires focus exclusively on the fields directly affected by health, health status questionnaires include physical, emotional, social, health and functional aspects. The use of the LCQ in evaluating patients with COPD will provide the clinician a general idea about the patients’ general quality of life.

### Limitations

The limitations of our study are the insufficient number of subjects in both study groups, the composition of the healthy subjects group that included mainly sedentary office workers and some of them had a history of smoking, no adjustment for key variables as socioeconomic status and comorbidities, study does not include COPD patients from every stage uniformly and the failure to use computerized dynamometers which provide more precise results of quadriceps muscle strength.

## Conclusions

In conclusion, this study showed that in a patient group with mainly moderate and severe COPD experience a loss of peripheral muscle strength and endurance, exercise capacity and general quality of life compared to healthy controls. Additionally, the effect of dyspnea perception and fatigue on general daily life activities were increased in comparison to healthy subjects; dyspnea perception and peripheral muscle weakness are related with the perception of fatigue. Coughing, which is an important symptom related with exacerbation and mortality in COPD patients, negatively affects quality of life by creating physical and psychosocial complications and this state also decreases general quality of life. The strength of this study is to show that multidimensional evaluations must be done in order to plan pulmonary rehabilitation programs for COPD patients. We think that showing the distinctive characters of assessment parameters for patients with COPD may guide the selection of the methods for evaluation and goal-appropriate treatment within pulmonary rehabilitation programs.

## Competing interests

The authors declare that they have no competing interests.

## Authors’ contributions

*ECK*: Data entry, critical interpretation of the results, manuscript preparation and revision. *SS*: Conception and design of the study, manuscript preparation and revision. *MS*: Data entry, critical interpretation and diccussion of the results, manuscript revision. *NVY*: Critical interpretation and diccussion of the results, manuscript revision. *DII*: Critical interpretation and discussion of the results, manuscript revision. *HA*: Critical interpretation and discussion of the results, manuscript revision. *ZA*: Data collection of patients with COPD, manuscript revision. *OO*: Data collection of healthy subjects, manuscript revision. *MBG:* Data analysis. *LC*: Clinical assessment of patients with COPD and redirection of patients eligible for study, manuscript revision. All authors read and approved the final manuscript.

## Pre-publication history

The pre-publication history for this paper can be accessed here:

http://www.biomedcentral.com/1471-2466/14/6/prepub
